# Efficacy of citicoline as a supplement in glaucoma patients: A systematic review

**DOI:** 10.1371/journal.pone.0291836

**Published:** 2023-09-28

**Authors:** Julia Prinz, Verena Prokosch, Hanhan Liu, Peter Walter, Matthias Fuest, Filippo Migliorini

**Affiliations:** 1 Department of Ophthalmology, RWTH Aachen University, Aachen, Germany; 2 Department of Ophthalmology, Faculty of Medicine and University Hospital of Cologne, Cologne, Germany; 3 Department of Orthopaedic and Trauma Surgery, RWTH Aachen University, Aachen, Germany; 4 Department of Orthopedics and Trauma Surgery, Academic Hospital of Bolzano (SABES-ASDAA), Bolzano, Italy; Irrua Specialist Teaching Hospital, NIGERIA

## Abstract

**Purpose:**

Glaucoma is a leading cause of irreversible blindness worldwide. Retinal ganglion cells (RGC), the neurons that connect the eyes to the brain, specifically die in glaucoma, leading to blindness. Elevated intraocular pressure (IOP) is the only modifiable risk factor, however, many patients progress despite excellent IOP control. Thus, alternative treatment strategies to prevent glaucoma progression are an unmet need. Citicoline has demonstrated neuroprotective properties in central neurodegenerative diseases. However, conclusive evidence of the effect of citicoline on glaucoma progression is missing. This systematic review investigates first-time the therapeutic potential of citicoline in glaucoma patients.

**Methods:**

The present study was conducted according to the PRISMA 2020 statement. PubMed, Web of Science, Google Scholar, and Embase were accessed in July 2023 to identify all clinical studies investigating the efficacy of citicoline on IOP, the mean deviation of the 24–2 visual field testing (MD 24–2), retinal nerve fibre layer (RNFL), and the pattern electroretinogram (PERG) P50-N95 amplitude in glaucoma patients. The risk of bias was assessed using the Review Manager 5.3 software (The Nordic Cochrane Collaboration, Copenhagen) and the Risk of Bias in Non-randomised Studies of Interventions (ROBINS-I) tool.

**Results:**

Ten studies were eligible for this systematic review, including 424 patients. The mean length of the follow-up was 12.1 ± 11.6 months. The overall risk of bias was low to moderate. The mean age of the patients was 56.7 years. There were no significant differences in the IOP, MD 24–2, RNFL, or PERG P50-N95 amplitude between patients receiving citicoline and the control group. There was no improvement from baseline to the last follow-up in IOP, MD 24–2, RNFL, or PERG P50-N95 amplitude.

**Conclusion:**

There is a lack of sufficient evidence to support that citicoline slows the progression of glaucoma.

## Introduction

Glaucoma is a leading cause of irreversible blindness [1], characterized by an irreversible loss of retinal ganglion cells (RGC) and their axons [2]. The global prevalence of glaucoma in the 40-80-year-old population is 3.5%. This is expected to increase [3]. RGC, the neurons that connect the eyes to the brain, fail to regenerate after damage, eventually leading to blindness [4]. Elevated intraocular pressure (IOP) is a main risk factor for glaucoma and the mainstay of treatment, however lowering IOP fails to halt the disease progression in many patients [5]. Novel strategies which halt RGC loss independently of IOP are desperately needed to prolong visual function in glaucoma [6]. Recent studies suggest that glaucomatous neuropathy does not only affect the retina and the optic nerve but also the lateral geniculate nucleus and the optic cortex [7]. Thus, neuroprotective treatments should target the optic nerve, RGC, and cerebral neurons [8, 9].

Citicoline (cytidine-5′-diphosphocholine, CDP-choline) is a naturally occurring endogenous precursor of the neurotransmitter acetylcholine and neuronal membrane components such as phosphatidylcholine or sphingomyelin [9]. Citicoline was demonstrated to delay the cognitive decline in patients with Alzheimer’s [10] and Parkinson’s disease [11], multiple sclerosis [12], cerebral ischemia [13], and traumatic brain injury [14]. Recently, the role of citicoline as a neuroprotective agent in various ophthalmologic conditions, including amblyopia [15], non-arteritic ischaemic optic neuropathy [16], corneal oxidative damage [17], and glaucoma [18] has been investigated. The neuroprotective effect of citicoline is attributed to its capacity to reduce glutamate excitotoxicity [19] and oxidative stress [20], to increase the neurotrophin level and improve the axoplasmic transport [21], to improve mitochondrial function [22], and to modulate the cerebral insulin signaling cascade [23].

Experimental studies on retinal cell cultures and animal models showed a protective effect of citicoline on glaucomatous damaged RGC [24, 25]. In patients with glaucoma, the use of citicoline as a supplement to the IOP-lowering medications has also been postulated. However, individual studies include small numbers of patients. Therefore, results from comparative studies are inconclusive [18, 26–30]. This systematic review aims to accumulate the existing evidence and to achieve a precise investigation of the efficacy of citicoline as an adjunctive therapy in glaucoma patients.

## Material and methods

### Eligibility criteria

All the clinical trials analysing the efficacy of citicoline as an adjunctive therapy in addition to any IOP-lowering medications in patients with glaucoma were accessed. Studies combining citicoline with other neuroprotective substances in the citicoline group were excluded. According to the authors language capabilities, articles in English, German, Italian, French, and Spanish were eligible. According to Oxford Centre of Evidence-Based Medicine [31], studies with level I-IV of evidence were considered. Abstracts, reviews, cross-over studies, comments, opinions, letters, and editorials were not included. Animals, *in vitro*, biomechanics, computational, and cadaveric studies were not eligible.

### Search strategy

This systematic review was conducted according to the Preferred Reporting Items for Systematic Reviews and Meta-Analyses: the 2020 PRISMA statement [32]. The initial search was guided according to the PICO algorithm:

- P (Population): glaucoma patients;
- I (Intervention): citicoline supplementation in combination with IOP-lowering medications;
- C (Comparison): control group (IOP-lowering medications only, IOP-lowering medications combined with placebo);
- O (Outcomes): IOP, visual field testing (MD 24–2), retinal nerve fibre layer (RNFL), pattern electroretinogram (PERG) P50-N95 amplitude

The following databases were accessed in July 2023: PubMed, Web of Science, Google Scholar, and Embase. No time constraints were used for the search. The following keywords were used in combination using the Boolean operators AND/OR: *glaucoma*, *open-angle glaucoma*, *primary open-angle glaucoma*, *OAG*, *POAG*, *normal tension glaucoma*, *NTG*, *angle closure glaucoma*, *pseudoexfoliative glaucoma*, *secondary glaucoma*, *pigmentary glaucoma*, *juvenile glaucoma*, *infantile glaucoma*, *aphakic glaucoma*, *phacogenic glaucoma*, *ciliary block glaucoma*, *neovascular glaucoma*, *drug-induced glaucoma*, *citicoline*, *CDP-choline*, *cytidine-5′-diphosphocholine*, *neuroprotection*, *intraocular pressure*, *pattern electroretinogram P50-N95 amplitude*, *PERG P50-N95*, *visual field*, *visual field 24–2*, *MD 24–2*, *retinal nerve fibre layer*, *RNFL*, *neuroretinal rim*.

### Selection and data collection

Two authors (XX; XX) independently performed the database search. All the resulting titles were screened and if suitable, the abstract was accessed. The full-texts of the abstracts which matched the topic were accessed. A cross reference of the bibliography of the full-text articles was also screened for inclusion. Disagreements were debated and the final decision was made by a third author (XX).

### Data items

Two authors (XX; XX) independently performed data extraction. The following data at baseline were extracted: author, year of publication and journal, length of the follow-up, number of patients, mean age of the patients, subtype of glaucoma, and the type of treatment with the related administration protocol. The outcomes of interest were the IOP, MD 24–2, RNFL, and the PERG P50-N95 amplitude, an objective parameter of the innermost retinal layer function, including ganglion cells and their fibers [33, 34].

### Methodological quality assessment

The risk of bias was evaluated according to the guidelines of the Cochrane Handbook for Systematic Reviews of Interventions [35]. The risk of bias of the included studies was assessed by two authors (XX; XX) independently. Disagreements were solved by a third author (XX). Randomized controlled trials (RCTs) were evaluated using the risk of bias of the software Review Manager 5.3 (The Nordic Cochrane Collaboration, Copenhagen) including selection, detection, performance, attrition, reporting, and other bias. Non-RCTs were assessed using the Risk of Bias in Non-randomised Studies of Interventions (ROBINS-I) tool.

### Statistical methods

All statistical analyses were performed by one author (XX) using Review Manager Software 5.3 (The Nordic Cochrane Centre, Copenhagen). The means and standard deviations were calculated for all descriptive statistics. The change from baseline to last follow-up was calculated using t-test analysis performed with the Statistical Package for Social Sciences (IBM SPSS Statistics for Windows, Version 25, Armonk, NY: IBM Corp.). The mean difference method was adopted with a 95% confidence interval (CI) and standard error (SE). Values of P < 0.05 were considered statistically significant.

## Results

### Study selection

**Fig 1:**
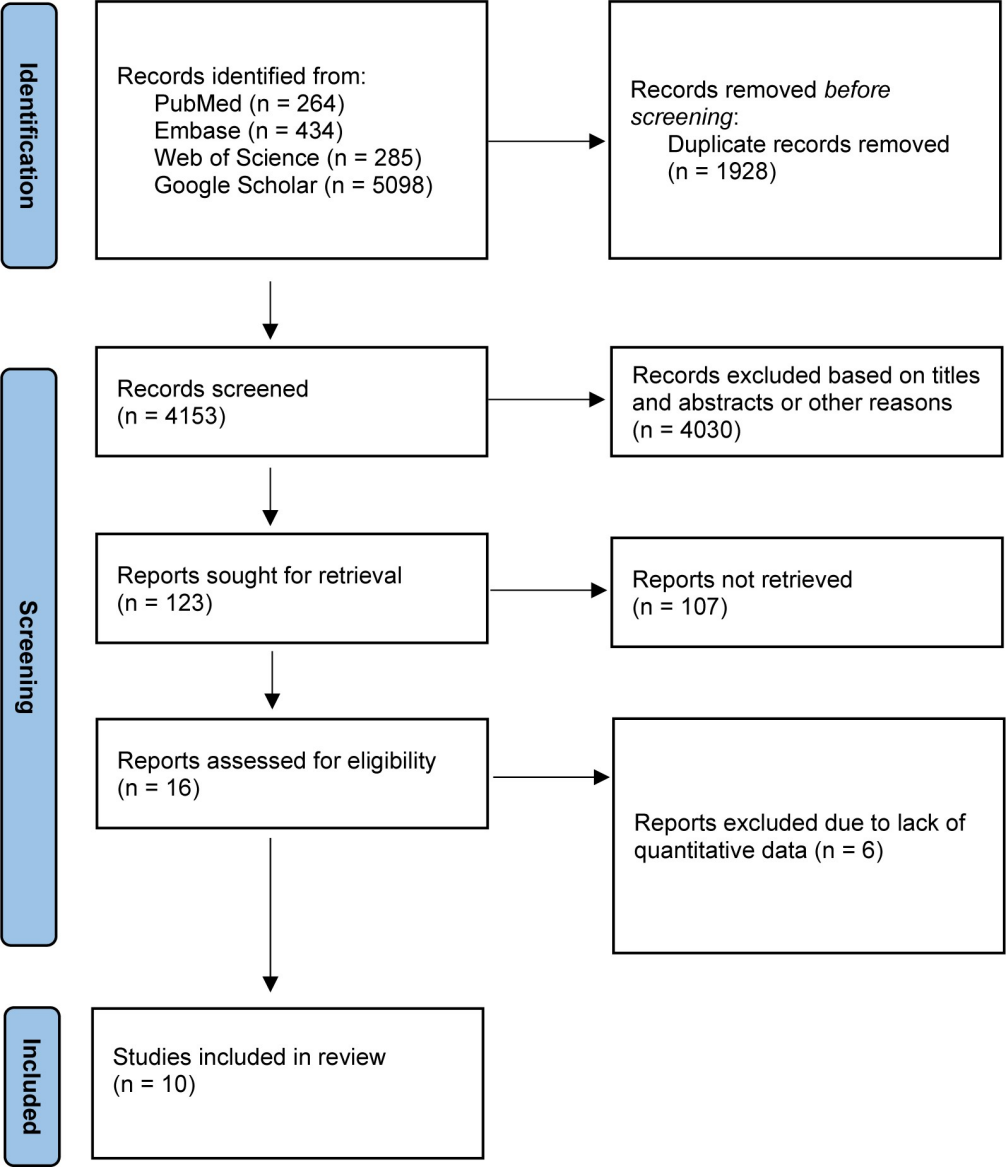
Flow chart of the literature search.

The literature search resulted in 6081 records from the databases PubMed, Embase, Web of Science, and Google Scholar (Fig 1). Of them, 1928 were excluded because of redundancy. Another 4030 were removed based on the titles and abstracts of the records. A further 107 studies were screened but not included in the systematic review because they did not meet the eligibility criteria. Another 6 studies did not report quantitative data under the endpoints of interest and were therefore excluded from our study. Finally, 10 studies were included in this systematic review, including 5 RCTs and 5 retrospective clinical studies. The literature search results are shown in Fig 1.

### Risk of bias assessment

**Fig 2:**
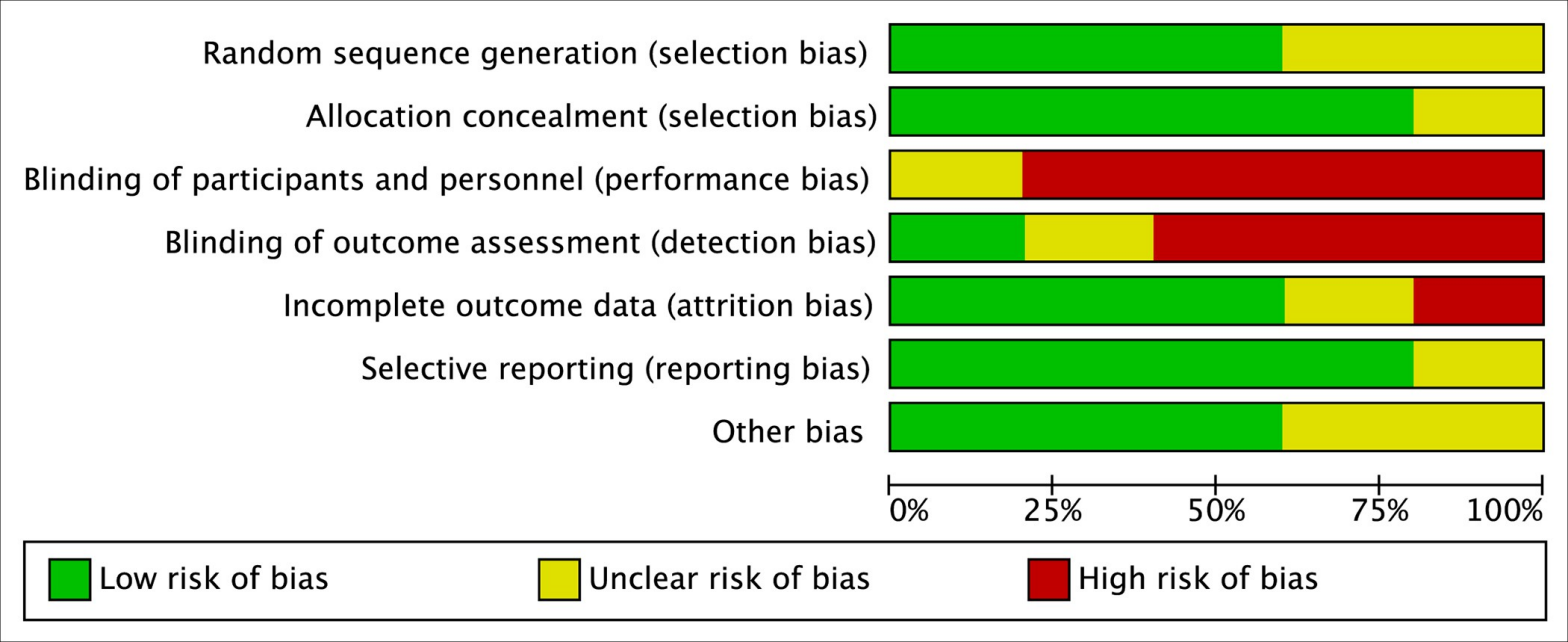
Risk of bias graph for randomized controlled studies.

The overall quality of the methodological assessment was low to moderate. Five studies (50%) were retrospective, and blinding was seldom performed. The authors’ judgement of each risk of bias item for the studies included is displayed in Table 1 for non-RCTs (ROBINS-I) and in Fig 2 for RCTs (risk of bias graph).

**Table 1:** Methodological quality assessment for non-randomized studies using the risk of bias in non-randomised studies of interventions (ROBINS-I) tool.

Author, year	Confounding	Participant selection	Classification of interventions	Deviations from intended intervention	Missing data	Measurement of outcomes	Selection of reported results	Overall risk of bias
Lanza et al. 2009 [27]	*Moderate*	*Moderate*	*Low*	*Low*	*Moderate*	*Low*	*Low*	*Moderate*
Ottobelli et al. 2013 [28]	*Moderate*	*Low*	*Moderate*	*Moderate*	*Low*	*Low*	*Low*	*Moderate*
Parisi et al. 2019 [29]	*High*	*Moderate*	*Moderate*	*Moderate*	*Low*	*Moderate*	*Moderate*	*Moderate*
Rejdak et al. 2003 [18]	*High*	*Moderate*	*Low*	*Low*	*Low*	*Low*	*Low*	*Moderate*
Sahin et al. 2022 [30]	*Moderate*	*Moderate*	*Low*	*Low*	*Low*	*Moderate*	*Low*	*Moderate*

### Study characteristics and results of individual studies

Data from 424 patients were collected. The mean length of the follow-up was 12.1 ± 11.6 months. The mean age of the patients was 56.7 ± 10.1 years. The generalities and patients’ characteristics of the included studies are shown in Table 2. The most common type of glaucoma was open-angle glaucoma (OAG, N = 419, 98.8%), followed by secondary glaucoma (N = 4, 0.9%), and normal tension glaucoma (N = 1, 0.2%). The secondary glaucoma cases were not further specified.

**Table 2:** Generalities and patients baseline of the included studies.

Author, et al. year	Journal	Study design	Treatment	Protocol	Follow-up (months)	Patients (n)	Mean age
Anton, 2022 [26]	*Life (Basel)*	RCT	Control group	Vitamin C	3	17	65.9
			Citicoline	1600 mg citicoline daily (orally) plus IOP-lowering medications	3	20	64.8
Lanza, 2009 [27]	*Front Pharmacol*	Retrospective	Citicoline	500 mg citicoline daily (orally) plus IOP-lowering medications	24	30	
			Control group	IOP-lowering medications	24	30	
Ottobelli, 2013 [28]	*Ophthalmologica*	Retrospective	Citicoline	500 mg citicoline daily (orally)	24	41	72.5
Parisi, 1999 [36]	*Ophthalmology*	RCT	Citicoline	1000 mg citicoline daily (intramuscularly) for 60 days plus IOP-lowering medications	12	10	45.6
			Citicoline	1000 mg citicoline daily (intramuscularly) for 120 days plus IOP-lowering medications	12	15	45.6
			Control group	placebo plus IOP-lowering medications	12	15	45.6
Parisi, 2015 [37]	*Graefes Arch Clin Exp Ophthalmol*	RCT	Citicoline	2% citicoline (eye drops) 3 times daily plus IOP-lowering medications	6	24	52.1
			Control group	IOP-lowering medications	6	23	52.7
Parisi, 2019 [29]	*Adv Ther*	Retrospective	Citicoline	2% citicoline (eye drops) 3 times daily plus IOP-lowering medications	4	12	52.6
Rejdak, 2003 [18]	*Med Sci Monit*	Retrospective	Citicoline	1000 mg citicoline daily (orally) plus IOP-lowering medications	2	21	
Roberti, 2014 [25]	*Indian J Ophthalmol*	RCT	Citicoline	2% citicoline eye drops 3 drops daily plus IOP-lowering medications	3	16	51.0
			Control group	IOP-lowering medications	3	18	51.0
Rossetti, 2020 [38]	*J Glaucoma*	RCT	Citicoline	500 mg citicoline daily (orally) plus IOP-lowering medications	36	40	74.0
			Control group	placebo plus IOP-lowering medications	36	38	71.4
Sahin, 2022 [30]	*Int J Ophthalmol*	Retrospective	Citicoline	250 mg citicoline daily (orally) plus IOP-lowering medications	4	27	52.2
			Control group	IOP-lowering medications	3	27	53.7

RCT: randomized controlled trial, IOP: intraocular pressure.

### Results of syntheses

Between-group comparability was found at baseline in the mean age, length of the follow-up, percentage of female patients, IOP, MD 24–2, RNFL, and PERG P50-N95 amplitude (Table 3).

**Table 3:** Baseline comparability of the citicoline and the control group.

Endpoint	Citicoline (N = 256)	Control (N = 168)	Mean difference	P
Follow-up (months)	11.8 ± 11.4	12.4 ± 12.9	0.6	0.9
Mean age (years)	56.7 ± 10.9	56.7 ± 9.8	0.0	1.0
Women (%)	54.1 ± 15.1	53.9 ± 2.7	-0.2	1.0
IOP (mmHg)	16.6 ± 4.1	17.1 ± 5.1	0.6	0.8
MD 24–2 (dB)	-6.8 ± 2.3	-7.3 ± 2.2	-0.5	0.8
RNFL (μm)	69.9 ± 6.9	72.2 ± 11.5	2.4	0.7
PERG P50-N95 amplitude (mVolt)	0.9 ± 0.2	1.0 ± 0.1	0.1	0.6

IOP: intraocular pressure, MD 24–2: mean deviation of the visual field testing 24–2, RNFL: retinal nerve fibre layer, PERG: pattern electroretinogram.

The use of citicoline did not demonstrate any improvement from baseline to the last follow-up in IOP (P = 0.3), MD 24–2 (P = 0.7), RNFL (P = 0.8), and PERG P50-N95 amplitude (P = 0.2, Table 4).

**Table 4:** Evaluation of the changes of the endpoints from baseline to the last follow-up.

Endpoint	baseline	last follow-up	Mean difference	P
IOP (mmHg)	16.6 ± 4.1	14.5 ± 0.6	-2.1	0.3
MD 24–2 (dB)	-6.8 ± 2.3	-7.4 ± 2.8	-0.5	0.7
RNFL (μm)	69.9 ± 6.9	72.2 ± 11.7	2.4	0.8
PERG P50-N95 amplitude (mVolt)	0.9 ± 0.2	1.2 ± 0.5	0.3	0.2

IOP: intraocular pressure, MD 24–2: mean deviation of the visual field testing 24–2, RNFL: retinal nerve fibre layer, PERG: pattern electroretinogram.

Compared to the control group, the administration of citicoline was not associated with a significant improvement in IOP, MD 24–2, RNFL, and PERG P50-N95 amplitude (Table 5).

**Table 5:** Comparison of citicoline *versus* the control group.

Endpoint	Citicoline (N = 256)	Control (N = 168)	Mean difference	P
IOP (mmHg)	14.5 ± 0.6	14.2 ± 1.2	0.3	0.6
MD 24–2 (dB)	-7.4 ± 2.8	-9.5 ± 1.6	2.1	0.4
RNFL (μm)	72.2 ± 1.7	71.8 ± 18.0	0.5	1.0
PERG P50-N95 amplitude (mVolt)	1.2 ± 0.5	1.0 ± 0.1	0.2	0.6

IOP: intraocular pressure, MD 24–2: mean deviation of the visual field testing 24–2, RNFL: retinal nerve fibre layer, PERG: pattern electroretinogram.

The subgroup comparison of the two groups over time did not demonstrate any superiority of citicoline *versus* the control group.

## Discussion

According to the main findings of the present systematic review, treatment with citicoline did neither lead to a significant reduction in IOP or MD 24–2, nor to a significant improvement in RNFL or the PERG P50-N95 amplitude in patients with glaucoma.

In the first reports investigating the effects of citicoline as a supplementary treatment to prevent glaucoma progression, citicoline was administered intramuscularly [39, 40], later orally [18, 26–28] and topically [25, 29, 38]. Intramuscular citicoline supplementation was associated with an improvement of visual evoked potential (VEP) and PERG in two RCTs [36, 41]. A long-term study reported the stabilization of visual field parameters in the citicoline group compared to the control group [40]. In this study, 1g citicoline was injected intramuscularly per day for 15 days every 6 months [40]. Rejdak et al. showed that an oral administration of citicoline (1g daily for 2 weeks) also led to a significant increase in the VEP amplitude and reduced VEP latency [18]. Parisi et al. found a significant improvement in retinal function evaluated by VEP, which partially subsided after 2 periods of a wash-out for 120-days following a daily administration of 1g citicoline intramuscularly and 1.6 g orally in 60 patients with ocular hypertension or open-angle glaucoma. Continuous or periodic administration of citicoline was necessary to provide a neuroprotective effect [42].

Recently, Carnevale et al. demonstrated that topically administered citicoline eye drops reach the vitreous in high concentrations in humans [43]. In a case-control study, citicoline was applied topically (citicoline 0.2 g eye drops, 3 times daily) in 24 patients with open-angle glaucoma [37]. All patients received beta-blocker monotherapy to control IOP, which was <18 mmHg during the study period as an inclusion criterion. After a treatment period of 4 months, a significant shortening of VEP P 100 implicit times and an increase of PERG N50-N95 and VEP N75-P100 amplitudes were observed [37]. However, citicoline’s duration of action is short, after a washout period of 2 months, no statistically significant changes compared to baseline were found [37].

The present study has several limitations. The limited number of clinical studies included for analysis represents the most important limitation. Furthermore, the retrospective design of 50% (5 of 10) of the included studies limits the evidence of the present study. All clinical studies investigating the effects of citicoline on the outcomes of interest were included, irrespective of the citicoline dose, administration route, or treatment duration. Given the limited quantitative data available for inclusion, no subgroup analysis was performed.

Recently, Garway-Heath et al. showed that a period of at least 24 months with 6 clustered visual field tests (3 at baseline and 3 at 24 months) was necessary to adequately monitor visual field progression [5, 44]. However, this follow-up scheme was not applied in any of the included studies. Therefore, the visual field results in this study should be interpreted considering this limitation.

Also, all patients receiving citicoline therapy continued their IOP-lowering medications therapy during the study period. Different IOP-lowering medications were used, resulting in heterogeneous groups. In addition, the heterogeneous length of the follow-up and treatment intervals as well as different control groups might also limit the reliability of the results. Continuous or periodic administration of citicoline might be necessary due to citicoline’s short duration of action [42]. Thus, conclusions from the present study must be analysed with caution. The studies included in this systematic review investigated the efficacy of citicoline as an adjuvant therapy in almost exclusively OAG patients; therefore, our results can not be generalized to all glaucoma subtypes. Future larger cohort studies are strongly warranted to further determine the impact of citicoline as adjunctive neuroprotective therapy in glaucoma patients or possibly preventive therapy in healthy patients with certain risk factors.

## Conclusion

There is a lack of evidence to suggest that citicoline led to a significant reduction in IOP or to a significant improvement in RGC preservation, the visual field, or the retinal function in glaucoma patients. Future randomized, prospective studies are needed to investigate the ideal treatment duration and dose.

## Supporting information

S1 Checklist (DOCX)
